# The Moderating Role of Social Network Size on Social Media Use and Self-Esteem: An Evolutionary Mismatch Perspective

**DOI:** 10.3389/fpsyg.2021.734206

**Published:** 2021-09-27

**Authors:** Amy J. Lim, Clement Lau, Norman P. Li

**Affiliations:** ^1^Discipline of Psychology, College of Science, Health, Engineering and Education, Murdoch University, Murdoch Singapore, Singapore, Singapore; ^2^School of Social Sciences, Singapore Management University, Singapore, Singapore

**Keywords:** social media use, social comparison, self-esteem, evolutionary mismatch, social network size

## Abstract

Existing meta-analyses have shown that the relationship between social media use and self-esteem is negative, but at very small effect sizes, suggesting the presence of moderators that change the relationship between social media use and self-esteem. Employing principles from social comparison and evolutionary mismatch theories, we propose that the social network sizes one has on social media play a key role in the relationship between social media use and self-esteem. In our study (*N* = 123), we showed that social media use was negatively related to self-esteem, but only when their social network size was within an evolutionarily familiar level. Social media use was not related to self-esteem when people’s social networks were at evolutionarily novel sizes. The data supported both social comparison and evolutionary mismatch theories and elucidated the small effect size found for the relationship between social media use and self-esteem in current literature. More critically, the findings of this study highlight the need to consider evolutionarily novel stimuli that are present on social media to better understand the behaviors of people in this social environment.

## Introduction

Popular social media platforms such as Facebook and Instagram have observed at least 50% of their users visiting the platforms on a daily basis (Smith and Anderson, 2018). Users typically spend a total of 2 h 25 min on social media each day, which can be equated to a full day of their waking hours each week (Datareportal, 2021). As virtual engagement with others on social media becomes an integral part of everyday life, the real-life consequences it carries for its users have become key public concerns and received notable research attention (e.g., Kim et al., 2009; Valenzuela et al., 2009; Morrison and Gore, 2010; Nabi et al., 2013; Neira and Barber, 2014; Sbarra et al., 2019)—one such area of research is its effects on self-esteem. While existing findings do show a negative relationship between social media use and self-esteem, the effect sizes found for this relationship are extremely small (Liu and Baumeister, 2016; Huang, 2017; Saiphoo et al., 2020). Researchers described the relationship between social media use and self-esteem as a “puzzling” one, accompanied with complicated conclusions (Liu and Baumeister, 2016). Using principles from social comparison and evolutionary mismatch theories, this paper aims to borrow an evolutionary lens in untangling the complex relationship between social media use and self-esteem.

### Social Comparison on Social Media

According to social comparison theory, people have an innate tendency to compare themselves to others (Festinger, 1954). In doing so, they derive at various outcomes, including an evaluation of themselves (Festinger, 1954), regulation of emotions and well-being (Taylor and Brown, 1988), and aspirations to improve their skills or abilities (Wood, 1989). Upward social comparison occurs when people compare themselves to others who are better than them; although upward social comparison motivates people to become more like their comparison target, it also causes dissatisfaction and lowers self-esteem (Emmons and Diener, 1985; Taylor and Lobel, 1989; Wheeler and Miyake, 1992). In contrast, downward social comparison occurs when people compare themselves to others who are worse-off than them and such comparison often leads to more positive self-evaluation and enhanced mood (Wills, 1981; Pyszczynski et al., 1985).

People are highly selective in what they present on social media (Mendelson and Papacharissi, 2010). They carefully curate the things they upload on social media that portrays the “perfect” aspects of their lives, such as flattering photographs, expensive goods, and personal successes (Siibak, 2009; Gonzales and Hancock, 2011; Blease, 2015). People also tend to present themselves positively on social media (Vogel and Rose, 2016). They typically upload content that best represents their ideal self (Rosenberg and Egbert, 2011), or a version of themselves that they believe will be best liked by others (Madden and Smith, 2010). As such, what results is a proliferation of profiles on social media suggesting that a large number of people are doing well and lead happy and perfect lives. On top of these, the “like” button provides further information about a person’s popularity and social capital (Kim and Lee, 2011; Vitak and Ellison, 2013). Collectively, these serve as social information that people take in and compare themselves against (Fox and Moreland, 2015).

While people engage in both upward and downward social comparisons when they use social media, existing evidence suggest that upward social comparisons are engaged more frequently than downward social comparisons. Through experiential sampling, where participants were monitored across 2 weeks, Kross et al. (2013) found that Facebook use was associated with declines in subjective well-being over time. Blease (2015) also proposed that depression, resulting from Facebook use, is likely to be brought about by the conspicuous amount of positive impressions people are exposed to from their Facebook friends, which opens up opportunities for comparison and escalates risk for negative appraisals. These studies suggest that the use of social media triggers upward social comparisons, or “harmful” social comparisons (Kross et al., 2013), which underlies the declines in subjective well-being and increased likelihood for depression.

With the constant exposure to information about how perfect the lives of others are, people consistently perceive that others are better off than oneself (Chou and Edge, 2012; de Vries and Kühne, 2015; Appel et al., 2016). Consequently, the constant upward social comparison that people engage in while using social media results in lowered self-appraisals or self-esteem (Vogel et al., 2014). Existing meta-analyses show support for a negative relationship between social media use and self-esteem, evidencing that increased social media use is associated with decreased self-esteem (Liu and Baumeister, 2016; Huang, 2017; Saiphoo et al., 2020). However, the effect sizes reported for the relationship between social media use and self-esteem are often very small [*r* = −0.09 by Liu and Baumeister (2016); *r* = −0.04 by Huang (2017); and *r* = −0.08 by Saiphoo et al. (2020)], suggesting the presence of moderators that account for the different relationships between these variables.

Higher effect sizes for the negative association between social media use and self-esteem were found for studies that assessed problematic social media use (i.e., addictive social media use) than those that measured the frequency of general social media use (Saiphoo et al., 2020). Studies that measured social and collective self-esteem, instead of global self-esteem, reported a positive relationship between social media use and social self-esteem (Valkenburg et al., 2017; Saiphoo et al., 2020). A recent study by Valkenburg et al. (2021), which employed a 3-week experience sampling design, showed that people differed in their susceptibility toward the content on social media (e.g., not receiving many likes), which contributes to the small effect size found between social media use and self-esteem People who were less susceptible to social media content reported smaller fluctuations in their self-esteem; in contrast, people who were more susceptible to social media were likely to experience bigger fluctuations in self-esteem that would have canceled each other out across time (Valkenburg et al., 2021).

In this paper, beyond measurement artifacts and individual differences, we turn our focus to the features of social media and propose that the amount of social information uniquely afforded by social media plays a significant role in determining the relationship between social media use and self-esteem. Employing an evolutionary mismatch perspective, we argue that novel features of social media—in particular, large social network sizes—influence the social comparison process such that greater social media use may not necessarily result in self-esteem loss.

### Evolutionary Mismatch and Social Media

The evolutionary mismatch perspective posits that our evolved psychological mechanisms, which are designed to be adaptive in ancestral environments, are not well-suited to handle novel elements within the modern context (see Li et al., 2018, 2020). A classic example of the evolutionary mismatch concerns our evolved preference for sweet and fatty foods. As sweet and fatty foods were higher in calories, the preference for these foods were adaptive in the ancestral environment where such caloric-rich food were scarce. However, in modern environments where there is an abundance of over-processed food and food that contain large amounts of manufactured sugar, this food preference leads people to overconsume sweet and fatty foods, more than what our physiological systems are designed to handle. Because our mechanisms did not evolve to process the unnaturally high levels of fats and sugar found in modern contexts, health conditions such as obesity and diabetes ensues (Gluckman and Hanson, 2006).

Similarly, social media is a modern feature that contains several evolutionarily novel elements that can potentially influence the functioning of our evolved psychological mechanisms. Of particular focus in this paper is its affordance for an evolutionarily novel large social network size. Most popular social media platforms allow registered members to create personal profiles and interact with other users. Registered members can seek other users out *via* a search engine, browse their profiles, and befriend them (Blease, 2015). This ease of befriending others contributes to the large “friend” networks people have on social media. The average adult Facebook user has 338 “friends”; beyond people who they actually are friends with in real life, this social network also comprises of people who are not close friends and people they have never met (Osman, 2021). However, humans have evolved to handle only a limited number of relationships (Tooby and Cosmides, 1996). Specifically, humans have evolved a neocortex size to maintain a network size of 150 individuals (Dunbar, 1998). This introduces a mismatch situation, which carries important implications for the psychological mechanisms governing social comparison.

As people are exposed to the “perfect” lives of others on social media, the evolved tendency to take in the social information and compare themselves to others results in self-esteem loss. Typically, the more one uses social media, the more social comparisons are engaged, and the more one feels worse about themselves. Moreover, on the surface, we might expect this to be even more true for networks with a greater vs. lesser number of people. Just as how our preference for sweet and fatty foods is hijacked by the modern environment, the social comparison process is hijacked by the large amount of social information introduced by large social network sizes, such that people are drawn into more social comparisons within larger networks. As such, on one hand, larger networks increase the occurrence for comparative social evaluation, which escalates the likelihood of one feeling more depressed and greater loss of self-esteem (e.g., Blease, 2015). Yet, a key evolutionary principal suggests otherwise. That is, given the natural limitations on humans’ ability to process network sizes, when social networks are beyond the size of 150 individuals, the enormous amount of available social information may be increasingly difficult for psychological mechanisms underlying social comparison to process. As such, on the other hand, for evolutionarily novel social network sizes that exceed 150 individuals, greater use of social media may not lead to greater loss of self-esteem.

### The Present Research

We began our research with the aim of understanding the negative but weak relationship between social media use and self-esteem. Using principles from social comparison theory and the evolutionary mismatch theory, we explore how social network size influences the relationship between social media use and self-esteem. Specifically, we predict that greater social media use is likely to be associated with lower levels of self-esteem when one’s social network size is within 150 individuals, the number of relationships we have evolved to handle. When social network sizes are larger than 150 individuals, we test the competing predictions: on one hand, with more targets for social comparison, greater use of social media is likely to result in greater self-esteem loss; on the other hand, the huge, evolutionarily novel amount of social information makes it difficult for psychological mechanisms underlying social comparison to process such that greater use of social media is not associated with low self-esteem.

## Materials and Methods

### Participants

A total of 123 participants were recruited through an Australian university’s subject pool system (106 females, *M*_*age*_ = 22.78, *SD*_*age*_ = 7.92). All participants indicated that they engage in at least one social media platform (*M* = 2.76, *SD* = 0.82), with Facebook (*N* = 111) and Instagram (*N* = 104) being the most used social media platforms. Participants reported having a mean of 1,186 friends (*SD* = 1,601) across all social media platforms that they engaged in.^1^

### Procedure

Upon providing informed consent, participants completed a series of questionnaires that measured their social media usage and self-esteem. Participants were also required to provide the number of friends they have across all the social media platforms they use. Finally, participants provided demographic details before completing the study.

### Materials

#### Social Media Use

Social media use was assessed with 10 items adapted from the Media and Technology Usage and Attitudes Scale (Rosen et al., 2013). Participants indicated the frequency of which they engaged in activities on social media; they responded to items such as “Post updates on your social media,” and “Browsed through profiles and photos” on a 10-point scale (1 = *never*, 10 = *all the time*). The items were averaged to form a single index for social media usage, where higher scores indicated more frequent usage (*M* = 4.83, SD = 1.18, α = 0.91).

#### Self-Esteem

Self-esteem was assessed using Rosenberg’s (1965) Self-esteem Scale. Participants responded to 10 items, such as “On the whole, I am satisfied with myself,” and “I feel that I’m a person of worth,” on a 4-point scale (1 = *Strongly disagree*, 4 = *Strongly agree*). Negatively worded items were reversed scored, and together, the 10 items were averaged to form a single index for self-esteem, where higher scores indicated higher levels of self-esteem (*M* = 2.82, SD = 0.56, α = 0.89).

#### Analytical Strategy

Descriptive statistics were provided for social media use, self-esteem, and number of friends. The assumption of normality was first assessed. Values for skewness and kurtosis for social media use (Skew = −1.26, Kurtosis = 1.67) and self-esteem (Skew = −0.08, Kurtosis = 0.22) were between −2 and +2, which were acceptable standards for a normal distribution (George and Mallery, 2010). For number of friends, the values for skewness and kurtosis were 3.46 and 15.19 respectively, indicating that this variable was not normally distributed. However, as we intended to convert number of friends into a categorical variable that reflects the different social network layers proposed by Dunbar (2011); Dunbar et al. (2015), we did not perform any other transformation of this variable to fit within acceptable standards of skewness and kurtosis. Univariate outliers were identified for social media use (*N* = 5), self-esteem (*N* = 1), and number of friends (*N* = 7). The subsequent moderation analysis was conducted with and without these univariate outliers.

As we are interested to examine number of friends in terms of evolutionarily familiar vs. evolutionarily novel levels (instead of number of friends *per se*), we transformed the number of friends participants reported they had across all their social media platforms into a categorical variable, which should ideally correspond to the social network layers identified by Dunbar (2011) and Dunbar et al. (2015). Dunbar (2011) and Dunbar et al. (2015) identified a mean network size of 150 individuals as a personal network, a mean network size of 500 individuals as a network characterized by acquaintances; beyond these, one’s social network of approximately 1,500 individuals is likely to consist of individuals one would merely recognize and not share meaningful relationships with. Through categorizing number of friends according to quartiles, we derived at four groups: participants with a social network size of 276 and below (small social network, *N* = 31, *M* = 114.26, *SD* = 91.85), participants with a social network size of 700 and below (medium social network, *N* = 31, *M* = 466.13, *SD* = 139.11), participants with a social network size of 1,500 and below (big social network, *N* = 32, *M* = 1,112.97, *SD* = 265.84), and participants above 1,500 (large social network, *N* = 29; *M* = 3,179.90, *SD* = 2,256.90). Although the cut-off values for the number of friends in small and medium social network groups are higher than those identified by Dunbar (2011) and Dunbar et al. (2015), researchers have recognized that there is wide variance around the mean network sizes (e.g., for the mean network size of 150, the lower and upper bounds are 100 and 250) (Dunbar, 2018) and are likely to be higher in an online context (Wellman, 2012). As such, the difference in values for social network sizes between our study and those identified by Dunbar (2011) and Dunbar et al. (2015) is unlikely to be of major concern.

To examine if the relationship between social media use and self-esteem differs at different social network sizes, we planned to conduct a moderation analysis. Prior to testing the moderation model, statistical assumptions relevant to a multiple regression analysis—that is, normality, linearity and homoscedasticity of residuals, and multicollinearity between predictors- was examined, and no assumptions violations were noted.

## Results

Table 1 displays the means, standard deviations, skewness, kurtosis, and intercorrelations of all the variables involved in this study. Correlation analysis indicated that social media use was not correlated to self-esteem (*r* = −0.08, *p* = 0.35), but social media use was positively associated with number of friends (*r* = 0.38, *p* < 0.01). Self-esteem was also not related to number of friends (*r* = 0.14, *p* = 0.12).

**TABLE 1:** Descriptive statistics and intercorrelations of all variables (*N* = 123).

Variables	1.	2.	3.
1. Social media use	−		
2. Self-esteem	–0.08	−	
3. Number of friends	0.38**	0.14	–
Mean	4.83	2.82	1,185.65
SD	1.18	0.56	1,601.18
Skew	–1.26	–0.08	3.46
Kurtosis	1.67	–0.07	15.26

**Correlation significant at *p* < 0.01.

A moderation analysis using PROCESS (Hayes, 2017) was conducted to examine if social network size moderated the relation between social media use and self-esteem. The four level categorical variable of social network size was dummy coded to reflect three vector codes (0’s and 1’s), with small social network size as the reference category. The moderation model accounted for significant unique variance in social media use, *R*^2^ = 0.20, *F* (7, 101) = 3.64, *p* < 0.01, *f*^2^ = 0.25.^2^ Social media use was associated negatively with self-esteem, *B* = −0.37, *t* (101) = −3.79, *p* < 0.01. Dummy coded variables, reflecting the difference between the small vs. medium social network size [*B* = −1.43, *t* (101) = −2.19, *p* = 0.03], and the difference between small and big social network size [*B* = −2.12, *t* (101) = −2.57, *p* = 0.01] was negatively related to self-esteem. The interaction term between social media use and social network size accounted for a significant 8.45% of the variance in self-esteem, *F* (3, 101) = 3.56, *p* = 0.02.

**FIGURE 1:**
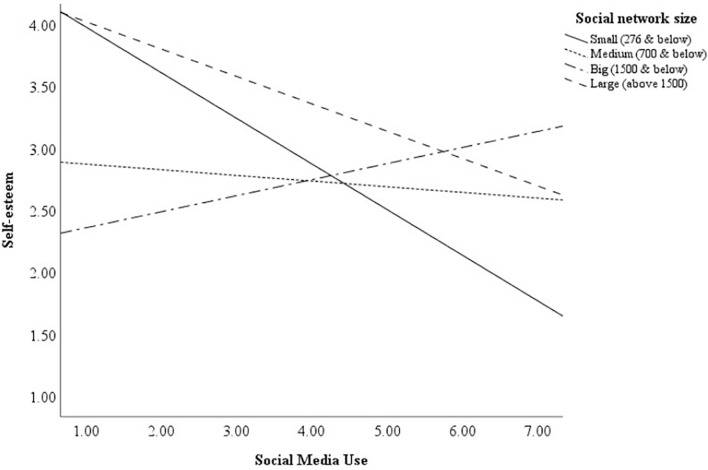
Moderation of social network size on social media use and self-esteem (*N* = 109).

Probing the moderation effect with simple slopes plot revealed that the relationship between social media use and self-esteem was significant only for small social network size, *B*_*small*_ = −0.37, *p* < 0.01, 95% CI [−0.56, −0.18] (Figure 1). The relationship between self-esteem and social media usage was not significant for medium [*B*_*medium*_ = −0.05, *p* = 0.64, 95% CI (−0.24, 0.15)], big [*B*_*big*_ = 0.13, *p* = 0.34, 95% CI (−0.14, 0.40)], and large social network sizes [*B*_*large*_ = −0.22, *p* = 0.19, 95% CI (−0.55, 0.11)]. These results showed that the number of friends one has on social media moderated the relation between one’s self-esteem and social media usage. Figure 1 demonstrates that at larger network sizes, the amount of social media use was not related to a person’s self-esteem.^3^

## Discussion

This study aimed to examine the negative, but weak, relationship between self-esteem and social media use. Employing principles from social comparison and evolutionary mismatch theories, we proposed that large social networks afforded by social media influences the functioning of psychological mechanisms involved in social comparison. Specifically, we argued that evolutionarily novel social network sizes (i.e., larger than 150 individuals) make it difficult for psychological mechanisms governing social comparison to process; and as such, the usual response, in which greater self-esteem loss results from increased social media use, is not produced. In this sense, greater social media use is likely to be associated with lower levels of self-esteem only when one’s social network size is evolutionarily familiar—that is, within 150 individuals—but not when social network sizes are larger than that. Our findings supported our prediction—social media use was negatively associated with self-esteem when social network size was small. Within this social network size, greater use of social media was associated with lower levels of self-esteem. In contrast, at larger social network sizes social media use was not significantly associated with self-esteem.

Consistent with existing meta-analyses, our results demonstrate that social media use share a negative relationship with self-esteem (Liu and Baumeister, 2016; Huang, 2017; Saiphoo et al., 2020). Beyond that, our study extends current literature by revealing that one’s social network size on social media moderates the relationship between social media use and self-esteem. Specifically, the characteristic of one’s social network size—whether it is evolutionarily familiar or novel—accounts for the different relationships between social media use and self-esteem, rather than absolute social network size *per se*. Kross et al. (2013) examined the moderating role number of Facebook friends between Facebook use and subjective well-being, and found that number of friends was not a significant moderator. Moreover, distinct from existing studies that have focused on individual differences, such as the tendency to engage in social comparisons (de Vries et al., 2018), and the susceptibility toward social media content (Valkenburg et al., 2021), this paper emphasizes on the amount of social information one is exposed to on social media in moderating the relationship between social media use and self-esteem. This emphasis on social information implies that people can potentially control their exposure to social information and its resulting outcomes, unlike the constrains present for individual differences (e.g., it is challenging to change one’s tendency to compare).

Furthermore, the current work extends both conceptual and empirical work on social comparison theory (Festinger, 1954). The application of social comparison theory to the context of social media has found an array of adverse consequences resulting from social media use. Across various social media platforms such as Facebook, people curate content to emphasize their most desirable traits and qualities and positive aspects of their lives (Manago et al., 2008; Vogel et al., 2014). As such, this perpetuates the persistent perception of being outnumbered by others who are succeeding in life. Coupled with our innate tendency to crave and digest social information, the exposure to such social information leads to comparative evaluations and negative appraisals about oneself (Ozimek and Bierhoff, 2020). Such upward online comparison more often causes people to feel inadequate, have poorer self-evaluations — which have been linked to various negative outcomes including depressive symptoms and negative emotions (Haferkamp and Krämer, 2011; Kalpidou et al., 2011; Feinstein et al., 2013; Blease, 2015). Our findings add on to this list of empirical work by demonstrating that greater social media is associated with lower levels of self-esteem.

The present research extends prior work on social comparison theory by revealing that social comparison can be influenced by evolutionary novel features of such media—the amount of social information that an individual is exposed to. While prior studies suggest that with larger social network sizes, people would engage in more social comparison (due to the presence of more comparison targets), and feel worse about themselves (Blease, 2015), our findings show that that is not the case. With an evolutionarily novel larger social network size, the use of social media is not associated with self-esteem. This suggests that at large social network sizes, social comparison affects people less, and hence, did not result in significant self-esteem loss.

Our findings also support evolutionary mismatch theory (Li et al., 2018, 2020), supporting the notion that inputs from the modern environment changes the normal functioning, and consequently, outputs, of ancestrally adaptive psychological mechanisms. Typically, the greater use of social media is accompanied by lower levels of self-esteem as people compare themselves with the flashy lifestyles and successes of others more. However, when social network sizes are larger than 150 individuals, this introduces a mismatch situation where the social information that is available is more than what we have evolved to handle (i.e., 150 individuals). And because large amounts of social information (when networks are beyond 150) are hard to process, greater social media use with large network sizes does not lead to greater loss of self-esteem. The results of our study reflects this pattern—social media use was associated with lower levels of self-esteem when social network size was evolutionarily familiar (i.e., within 150 individuals). On a broader level, this demonstrates that evolutionarily novel social network sizes affect the psychological output of the social comparison process.

Our results indicated that self-esteem levels were higher when social network sizes were larger. This could be due to people perceiving their relational values to be higher when their social network sizes are larger. According to the sociometer theory, self-esteem acts as a gauge to an individual’s relational value (Leary et al., 1995; Leary, 2005). Relational value refers to the degree to which one perceives their relationships with others is important and valuable (Leary, 2001). Existing studies have consistently demonstrated that one’s relational value is associated to their self-esteem; when people were made to believe that they possessed low relational value, through manipulations such as knowing others did not desire to interact with them or were excluded from groups, their self-esteem dropped (Leary et al., 1995; Leary, 2005). With a larger social network, coupled with our inability to distinguish real from virtual friends (Kanazawa, 2002), it leads to the perception that one had more “friends” and hence, a higher relational value. This would offer an explanation to the higher levels of self-esteem observed when one’s social network size was larger.

### Limitations and Future Directions

Our work is far from conclusive and poses questions for future work. Within the evolutionary framework, general intelligence may have evolved to solve evolutionarily novel problems (Kanazawa, 2010). This implies that the evolutionary constraints on the human brain proposed by the mismatch theory may be less strong among more intelligent than less intelligent individuals as they are more able to comprehend and deal with evolutionarily novel entities and situations (e.g., Kanazawa and Li, 2015). Given the evolutionarily novel nature of social media and large social networks, it is likely that intelligence may play an important role in influencing the effects of large social network sizes. For instance, more intelligent people may be more likely to be able to process the inputs of group sizes larger than the evolutionarily familiar limit of 150, and hence, engage in social comparisons as they would for network sizes of 150 individuals. However, it is also possible that they may be more able to perceive that these social networks consist of people who are not real (i.e., virtual friends) and not have their self-esteem affected in the first place. As such, future directions should examine the effects of intelligence on social media use.

Similar to existing studies that had examined the effect of social media use on subjective well-being (Kross et al., 2013) and depression (Blease, 2015), we proposed that social comparisons underlie the relationship between social media use and self-esteem, and that upward social comparisons tend to be made when people are engaged in social media, which would account the negative relationship observed between social media use and self-esteem (Liu and Baumeister, 2016; Huang, 2017; Saiphoo et al., 2020). While our findings show support for this negative relationship, social comparisons were not directly measured in this paper—we are only able to infer the social comparisons that could have taken place based on self-esteem, which would not accurately elucidate the social comparison process. Moreover, the type of social comparison influences self-esteem differently; while upward social comparisons result in low self-esteem, downward social comparison can boost self-esteem (Vogel et al., 2014). Future studies should explicitly assess the type and frequency of social comparisons people make when they engage in social media. Examining the social comparison process would not only provide evidence for the type of social comparisons people make when using social media, it would also shed light on how exactly large social networks affect the functioning of psychological mechanism governing social comparisons.

The analytic approach employed in this paper allows us to draw inferences about the association between naturally occurring levels of social media use and self-esteem, but it is not conclusive of the definitive causal relations between them. As such, an alternative interpretation to the findings in this paper is that self-esteem is also likely to influence social media use. Social media typically offers users opportunities for self-disclosure, feedback validation, and relationship development (Stern, 2004; Boyd, 2008; Reich et al., 2012). With these opportunities, social media was deemed to be particularly useful for individuals with lower levels of self-esteem who face difficulties in social situations in real life. Social media allows them to compensate their need for social interactions by allowing them to expand their social capital (Forest and Wood, 2012; Błachnio et al., 2013, 2016) and social snack through photos, representational reminders of social connections, and parasocial relationships (see Gardner et al., 2005). Thus, experiments manipulating social media use, and the number of friends one has, would be required to establish causal relationship between social media use and self-esteem. That said, these experiments would have to be carefully set up as participants should still feel socially connected after the possible manipulations (e.g., manipulated social profiles) in order to elicit meaningful social comparisons.

Having used a university’s psychology subject pool system, we recognize some of the shortcomings that accompany this sample. Participants in our participants were predominantly women who may be more influenced by the cues on social media than men. Compared to men, they tend to internalize media-promoted ideals to a higher degree (Knauss et al., 2007) and are more oriented to the activities of others when using social media (Steinsbekk et al., 2021). Studies also show that women were more likely to have negative emotional responses and experience depressive symptoms than men when using social media (Fleuriet et al., 2014; Kelly et al., 2018). As such, the effect of social media use on self-esteem is likely to be more pronounced in the current sample than if it was from a more gender-balanced sample. To this end, future studies may want to consider including non-binary gender measures to derive at more accurate conclusions for the effects of social media use (Cameron and Stinson, 2019). Additionally, participants were categorized into groups according to the quartiles of the number of friends they reported they had across all their social media platforms. While we observed significant findings for the interaction term and simple slopes, the number of participants in each group is considered small (roughly 30 per group). Hence, a larger sample size in future studies would provide greater confidence to the findings of this paper.

Furthermore, the amount of social information one is exposed to on social media is inferred from the number of friends one has in this study, which may not be a nuanced enough measure. The exposure to social information could be different depending on specific behaviors and the types of activities people engage in on social media. For instance, they may spend more time curating their profiles and working on their own posts than reading and interacting with those of others, and this implies that they would be less exposed to social information regardless of the number of friends they have on social media. As such, it would be beneficial for future studies to breakdown social information exposure through the different ways people spend their time on social media.

## Conclusion

This study aimed to examine the negative, but weak, relationship between social media use and self-esteem. Employing social comparison theory and an evolutionary mismatch perspective, we found that people’s social network size on social media moderated the relationship between social media use and self-esteem. Specifically, we found that greater social media use was associated with lower self-esteem only when social network size was evolutionarily familiar (i.e., within 150 individuals). When social network sizes were evolutionarily novel (i.e., social network sizes larger than 150 individuals), social media use was not associated with self-esteem. Our findings provide empirical evidence for a mismatch between the large social network sizes on social media and psychological mechanisms governing social comparison processes.

## Data Availability Statement

The raw data supporting the conclusions of this article will be made available by the authors upon request, without undue reservation.

## Ethics Statement

The studies involving human participants were reviewed and approved by Murdoch University Research Ethics with the following approval reference number: 2019/019. The patients/participants provided their written informed consent to participate in this study.

## Author Contributions

AL conceptualised the research idea, collected and analyzed the data, and drafted the manuscript. CL conducted initial data analyses and contributed to the literature review. NL contributed to the conceptualization of the research idea. All authors read, edited, and approved the final manuscript and agreed to be accountable for the content of this article.

## Conflict of Interest

The authors declare that the research was conducted in the absence of any commercial or financial relationships that could be construed as a potential conflict of interest.

## Publisher’s Note

All claims expressed in this article are solely those of the authors and do not necessarily represent those of their affiliated organizations, or those of the publisher, the editors and the reviewers. Any product that may be evaluated in this article, or claim that may be made by its manufacturer, is not guaranteed or endorsed by the publisher.

## Footnotes

1 On average, participants reported a mean of 482 friends (*SD* = 749) per social media platform.

2 We conducted a sensitivity power analysis using G-Power, which indicated that a minimum effect size of *f*^2^ = 0.12 is required for a total sample size of 109 to detect a power of 0.80 at α = 0.05.

3 The moderation analysis reported here excluded the univariate outliers. A similar analysis was conducted without removing the univariate outliers and similar results were found. Although the interaction term only approached significance, *F* (3, 115) = 1.77, *p* = 0.16, a significant negative relationship between social media use and self-esteem was evident only when social network size was small, *B*_*s**m**a**l**l*_ = −0.20, *p* < 0.01, 95% CI [−0.32, −0.07].

## References

[B1] Appel H. Gerlach A. L. Crusius J. (2016). The interplay between Facebook use, social comparison, envy, and depression. *Curr. Opin. Psychol.* 9 44–49. 10.1016/j.copsyc.2015.10.006

[B2] Błachnio A. Przepiórka A. Rudnicka P. (2013). Psychological determinants of using Facebook: a research review. *Int. J. Hum. Comput. Interact.* 29 775–787. 10.1080/10447318.2013.780868

[B3] Błachnio A. Przepiorka A. Rudnicka P. (2016). Narcissism and self-esteem as predictors of dimensions of Facebook use. *Pers. Individ. Dif.* 90 296–301. 10.1016/j.paid.2015.11.018

[B4] Blease C. R. (2015). Too many ‘friends,’too few ‘likes’? Evolutionary psychology and ‘Facebook depression’. *Rev. Gen. Psychol.* 19 1–13. 10.1037/gpr0000030

[B5] Boyd D. (2008). Facebook’s privacy trainwreck: exposure, invasion, and social convergence. *Convergence* 14 13–20. 10.1177/1354856507084416

[B6] Cameron J. J. Stinson D. A. (2019). Gender (mis)measurement: guidelines for respecting gender diversity in psychological research. *Soc. Personal. Psychol. Compass.* 13:e12506. 10.1111/spc3.12506

[B7] Chou H. T. G. Edge N. (2012). “They are happier and having better lives than I am”: the impact of using Facebook on perceptions of others’ lives. *Cyberpsychol. Behav. Soc. Netw.* 15 117–121. 10.1089/cyber.2011.0324 22165917

[B8] Datareportal (2021). *Global Social Media Stats.* Available online at: https://datareportal.com/reports/digital-2021-global-overview-report. (accessed June 30, 2021)

[B9] de Vries D. A. Kühne R. (2015). Facebook and self-perception: individual susceptibility to negative social comparison on Facebook. *Pers. Individ. Dif.* 86 217–221. 10.1016/j.paid.2015.05.029

[B10] de Vries D. A. Möller A. M. Wieringa M. S. Eigenraam A. W. Hamelink K. (2018). Social comparison as the thief of joy: emotional consequences of viewing strangers’ Instagram posts. *Media Psychol.* 21 222–245. 10.1080/15213269.2016.1267647

[B11] Dunbar R. (2011). How many “friends” can you really have? *IEEE Spectr.* 48 81–83. 10.1109/MSPEC.2011.5783712

[B12] Dunbar R. I. (1998). The social brain hypothesis. *Evol. Anthropol.* 6 178–190. 10.1002/(SICI)1520-6505(1998)6:5<178::AID-EVAN5>3.0.CO;2-8

[B13] Dunbar R. I. (2018). The anatomy of friendship. *Trends Cogn. Sci.* 22 32–51. 29273112 10.1016/j.tics.2017.10.004

[B14] Dunbar R. I. Arnaboldi V. Conti M. Passarella A. (2015). The structure of online social networks mirrors those in the offline world. *Soc. Netw.* 43 39–47. 10.1016/j.socnet.2015.04.005

[B15] Emmons R. A. Diener E. (1985). Personality correlates of subjective well-being. *Pers. Soc. Psychol. Bull.* 11 89–97. 10.1177/0146167285111008

[B16] Feinstein B. A. Hershenberg R. Bhatia V. Latack J. A. Meuwly N. Davila J. (2013). Negative social comparison on Facebook and depressive symptoms: rumination as a mechanism. *Psychol. Pop. Media Cult.* 2:161. 10.1037/a0033111 PMC3907111 24490122

[B17] Festinger L. (1954). A theory of social comparison processes. *Hum. Relat.* 7 117–140. 10.1177/001872675400700202

[B18] Fleuriet C. Cole M. Guerrero L. K. (2014). Exploring Facebook: attachment style and nonverbal message characteristics as predictors of anticipated emotional reactions to Facebook postings. *J. Nonverbal. Behav.* 38 429–450. 10.1007/s10919-014-0189-x

[B19] Forest A. L. Wood J. V. (2012). When social networking is not working: individuals with low self-esteem recognize but do not reap the benefits of self-disclosure on Facebook. *Psychol. Sci.* 23 295–302. 10.1177/0956797611429709 22318997

[B20] Fox J. Moreland J. J. (2015). The dark side of social networking sites: an exploration of the relational and psychological stressors associated with Facebook use and affordances. *Comput. Hum. Behav.* 45 168–176. 10.1016/j.chb.2014.11.083

[B21] Gardner W. L. Pickett C. L. Knowles M. (2005). “Social snacking and shielding: using social symbols, selves, and surrogates in the service of belonging needs,” in *The Social Outcast: Ostracism, Social Exclusion, Rejection, and Bullying*, eds Williams K. D. Forgas J. P. von Hippel W. (New York, NY: Psychology Press), 227–242.

[B22] George D. Mallery P. (2010). *SPSS for Windows Step by Step. A Simple Study Guide and Reference (10. Baskı).* Boston, MA: Pearson Education, Inc.

[B23] Gluckman P. D. Hanson M. A. (2006). *The Developmental Origins of Health and Disease. In Early Life Origins of Health and Disease.* Boston, MA: Springer. 10.1017/CBO9780511544699

[B24] Gonzales A. L. Hancock J. T. (2011). Mirror, mirror on my Facebook wall: effects of exposure to Facebook on self-esteem. *Cyberpsychol. Behav. Soc. Netw.* 14 79–83. 10.1089/cyber.2009.0411 21329447

[B25] Haferkamp N. Krämer N. C. (2011). Social comparison 2.0: examining the effects of online profiles on social-networking sites. *Cyberpsychol. Behav. Soc. Netw.* 14 309–314. 10.1089/cyber.2010.0120 21117976

[B26] Hayes A. F. (2017). *Introduction to Mediation, Moderation, and Conditional Process Analysis: A Regression-Based Approach.* New York, NY: Guilford publications.

[B27] Huang C. (2017). Time spent on social network sites and psychological well-being: a meta-analysis. *Cyberpsychol. Behav. Soc. Netw.* 20 346–354. 10.1089/cyber.2016.0758 28622031

[B28] Kalpidou M. Costin D. Morris J. (2011). The relationship between Facebook and the well-being of undergraduate college students. *Cyberpsychol. Behav. Soc. Netw.* 14 183–189. 10.1089/cyber.2010.0061 21192765

[B29] Kanazawa S. (2002). Bowling with our imaginary friends. *Evol. Hum. Behav.* 23 167–171. 10.1016/S1090-5138(01)00098-8

[B30] Kanazawa S. (2010). Evolutionary psychology and intelligence research. *Am. Psychol.* 65 279–289. 10.1037/a0019378 20455621

[B31] Kanazawa S. Li N. P. (2015). Happiness in modern society: why intelligence and ethnic composition matter. *J. Res. Pers.* 59 111–120. 10.1016/j.jrp.2015.06.004

[B32] Kelly Y. Zilanawala A. Booker C. Sacker A. (2018). Social media use and adolescent mental health: findings from the UK Millennium Cohort Study. *EClinicalMedicine* 6 59–68. 10.1016/j.eclinm.2018.12.005 31193561 PMC6537508

[B33] Kim J. LaRose R. Peng W. (2009). Loneliness as the cause and the effect of problematic internet use: the relationship between internet use and psychological well-being. *Cyberpsychol. Behav.* 12 451–455. 10.1089/cpb.2008.0327 19514821

[B34] Kim J. Lee J. E. R. (2011). The Facebook paths to happiness: effects of the number of Facebook friends and self-presentation on subjective well-being. *Cyberpsychol. Behav. Soc. Netw.* 14 359–364. 10.1089/cyber.2010.0374 21117983

[B35] Knauss C. Paxton S. J. Alsaker F. D. (2007). Relationships amongst body dissatisfaction, internalisation of the media body ideal and perceived pressure from media in adolescent girls and boys. *Body Image* 4 353–360. 10.1016/j.bodyim.2007.06.007 18089281

[B36] Kross E. Verduyn P. Demiralp E. Park J. Lee D. S. Lin N. (2013). Facebook use predicts declines in subjective well-being in young adults. *PLoS One* 8:e69841. 10.1371/journal.pone.0069841 23967061 PMC3743827

[B37] Leary M. R. (2001). “Towards a conceptualization of interpersonal rejection,” in *Interpersonal Rejection*, ed. Leary M. R. (New York, NY: Oxford University Press), 3–20. 10.1093/acprof:oso/9780195130157.003.0001

[B38] Leary M. R. (2005). Sociometer theory and the pursuit of relational value: getting to the root of self-esteem. *Eur. Rev. Soc. Psychol.* 16 75–111. 10.1080/10463280540000007

[B39] Leary M. R. Tambor E. S. Terdal S. K. Downs D. L. (1995). Self-esteem as an interpersonal monitor: the sociometer hypothesis. *J. Pers. Soc. Psychol.* 68:518. 10.1037/0022-3514.68.3.518

[B40] Li N. P. van Vugt M. Colarelli S. M. (2018). The evolutionary mismatch hypothesis: implications for psychological science. *Curr. Dir. Psychol. Sci.* 27 38–44. 10.1177/0963721417731378

[B41] Li N. P. Yong J. C. Van Vugt M. (2020). Evolutionary psychology’s next challenge: solving modern problems using a mismatch perspective. *Evol. Behav. Sci.* 14 362–367. 10.1037/ebs0000207

[B42] Liu D. Baumeister R. F. (2016). Social networking online and personality of self-worth: a meta-analysis. *J. Res. Pers.* 64 79–89. 10.1016/j.jrp.2016.06.024

[B43] Madden M. Smith A. (2010). *Reputation Management and Social Media.* Washington, DC: Pew Internet & American Life Project.

[B44] Manago A. M. Graham M. B. Greenfield P. M. Salimkhan G. (2008). Self-presentation and gender on MySpace. *J. Appl. Dev. Psychol.* 29 446–458. 10.1016/j.appdev.2008.07.001

[B45] Mendelson A. L. Papacharissi Z. (2010). “Look at us: collective narcissism in college student Facebook photo galleries,” in *A Networked Self: Identity, Community?and?Culture?on?Social?Network?Sites*, ed. Papacharissi Z. (New York, NY: Routledge), 259-281. 10.4324/9780203876527-20

[B46] Morrison C. M. Gore H. (2010). The relationship between excessive Internet use and depression: a questionnaire-based study of 1,319 young people and adults. *Psychopathology* 43 121–126. 10.1159/000277001 20110764

[B47] Nabi R. L. Prestin A. So J. (2013). Facebook friends with (health) benefits? Exploring social network site use and perceptions of social support, stress, and well-being. *Cyberpsychol. Behav. Soc. Netw.* 16 721–727. 10.1089/cyber.2012.0521 23790356

[B48] Neira B. C. J. Barber B. L. (2014). Social networking site use: linked to adolescents’ social self−concept, self−esteem, and depressed mood. *Aust. J. Psychol.* 66 56–64. 10.1111/ajpy.12034

[B49] Osman M. (2021). *Wild and Interesting Facebook Statistics and Facts (2021). Kinsta.* Available online at: https://kinsta.com/blog/facebook-statistics/ (accessed January 3, 2021).

[B50] Ozimek P. Bierhoff H. W. (2020). All my online-friends are better than me–three studies about ability-based comparative social media use, self-esteem, and depressive tendencies. *Behav. Inf. Technol.* 39 1110–1123. 10.1080/0144929X.2019.1642385

[B51] Pyszczynski T. Greenberg J. LaPrelle J. (1985). Social comparison after success and failure: biased search for information consistent with a self-serving conclusion. *J. Exp. Soc. Psychol.* 21 195–211. 10.1016/0022-1031(85)90015-0

[B52] Reich S. M. Subrahmanyam K. Espinoza G. (2012). Friending, IMing, and hanging out face-to-face: overlap in adolescents’ online and offline social networks. *Dev. Psychol.* 48:356. 10.1037/a0026980 22369341

[B53] Rosen L. D. Whaling K. Carrier L. M. Cheever N. A. Rokkum J. (2013). The media and technology usage and attitudes scale: an empirical investigation. *Comput. Hum. Behav.* 29 2501–2511. 10.1016/j.chb.2013.06.006 25722534 PMC4338964

[B54] Rosenberg J. Egbert N. (2011). Online impression management: personality traits and concerns for secondary goals as predictors of self-presentation tactics on Facebook. *J. Comput. Mediat. Commun.* 17 1–18. 10.1111/j.1083-6101.2011.01560.x

[B55] Rosenberg M. (1965). *Society and the Adolescent Self-Image.* Princeton, NJ: Princeton University Press. 10.1515/9781400876136

[B56] Saiphoo A. N. Halevi L. D. Vahedi Z. (2020). Social networking site use and self-esteem: a meta-analytic review. *Pers. Individ. Dif.* 153:109639. 10.1016/j.paid.2019.109639

[B57] Sbarra D. A. Briskin J. L. Slatcher R. B. (2019). Smartphones and close relationships: the case for an evolutionary mismatch. *Perspect. Psychol. Sci.* 14 596–618. 10.1177/1745691619826535 31002764

[B58] Siibak A. (2009). Constructing the self through the photo selection-visual impression management on social networking websites. *Cyberpsychology* 3:1.

[B59] Smith A. Anderson M. (2018). *Social Media Use in 2018. Pew Research Center.* Available online at: https://www.pewresearch.org/internet/2018/03/01/social-media-use-in-2018/ (accessed March 1, 2018).

[B60] Steinsbekk S. Wichstrøm L. Stenseng F. Nesi J. Hygen B. W. Skalická V. (2021). The impact of social media use on appearance self-esteem from childhood to adolescence–A 3-wave community study. *Comput. Hum. Behav.* 114:106528. 10.1016/j.chb.2020.106528

[B61] Stern S. R. (2004). Expressions of identity online: prominent features and gender differences in adolescents’ World Wide Web home pages. *J. Broadcast. Electron. Media* 48 218–243. 10.1207/s15506878jobem4802_4

[B62] Taylor S. E. Brown J. D. (1988). Illusion and well-being: a social psychological perspective on mental health. *Psychol. Bull.* 103 193–210. 10.1037/0033-2909.103.2.193 3283814

[B63] Taylor S. E. Lobel M. (1989). Social comparison activity under threat: downward evaluation and upward contacts. *Psychol. Rev.* 96 569–575. 10.1037/0033-295X.96.4.569 2678204

[B64] Tooby J. Cosmides L. (1996). Friendship and the banker’s paradox: other pathways to the evolution of adaptations for altruism. *Proc. Br. Acad.* 88 119–144.

[B65] Valenzuela S. Park N. Kee K. F. (2009). Is there social capital in a social network site?: facebook use and college students’ life satisfaction, trust, and participation. *J. Comput. Mediat. Commun.* 14 875–901. 10.1111/j.1083-6101.2009.01474.x

[B66] Valkenburg P. Beyens I. Pouwels J. L. van Driel I. I. Keijsers L. (2021). Social media use and adolescents’ self-esteem: heading for a person-specific media effects paradigm. *J. Commun.* 71 56–78. 10.1093/joc/jqaa039

[B67] Valkenburg P. M. Koutamanis M. Vossen H. G. (2017). The concurrent and longitudinal relationships between adolescents’ use of social network sites and their social self-esteem. *Comput. Hum. Behav.* 76 35–41. 10.1016/j.chb.2017.07.008 29104364 PMC5608942

[B68] Vitak J. Ellison N. B. (2013). ‘There’s a network out there you might as well tap’: exploring the benefits of and barriers to exchanging informational and support-based resources on Facebook. *New Media Soc.* 15 243–259. 10.1177/1461444812451566

[B69] Vogel E. A. Rose J. P. (2016). Self-reflection and interpersonal connection: making the most of self-presentation on social media. *Transl. Issues Psychol. Sci.* 2 294–302. 10.1037/tps0000076

[B70] Vogel E. A. Rose J. P. Roberts L. R. Eckles K. (2014). Social comparison, social media, and self-esteem. *Psychol. Pop. Media Cult.* 3:206. 10.1037/ppm0000047

[B71] Wellman B. (2012). Is Dunbar’s number up? *Br. J. Psychol.* 103 174–176. 10.1111/j.2044-8295.2011.02075.x 22506743

[B72] Wheeler L. Miyake K. (1992). Social comparison in everyday life. *J. Pers. Soc. Psychol.* 62 760–773. 10.1037/0022-3514.62.5.760

[B73] Wills T. A. (1981). Downward comparison principles in social psychology. *Psychol. Bull.* 90 245–271. 10.1037/0033-2909.90.2.245

[B74] Wood J. V. (1989). Theory and research concerning social comparisons of personal attributes. *Psychol. Bull.* 106 231–248. 10.1037/0033-2909.106.2.231

